# Information and Communication Technologies for the Dissemination of Clinical Practice Guidelines to Health Professionals: A Systematic Review

**DOI:** 10.2196/mededu.6288

**Published:** 2016-11-30

**Authors:** Gino De Angelis, Barbara Davies, Judy King, Jessica McEwan, Sabrina Cavallo, Laurianne Loew, George A Wells, Lucie Brosseau

**Affiliations:** ^1^ School of Rehabilitation Sciences Faculty of Health Sciences University of Ottawa Ottawa, ON Canada; ^2^ School of Nursing Faculty of Health Sciences University of Ottawa Ottawa, ON Canada; ^3^ Telfer School of Management University of Ottawa Ottawa, ON Canada; ^4^ School of Epidemiology, Public Health and Preventive Medicine University of Ottawa Ottawa, ON Canada

**Keywords:** health information technologies, electronic mail, email, Web 2.0, practice guidelines, health professions, information dissemination

## Abstract

**Background:**

The transfer of research knowledge into clinical practice can be a continuous challenge for researchers. Information and communication technologies, such as websites and email, have emerged as popular tools for the dissemination of evidence to health professionals.

**Objective:**

The objective of this systematic review was to identify research on health professionals’ perceived usability and practice behavior change of information and communication technologies for the dissemination of clinical practice guidelines.

**Methods:**

We used a systematic approach to retrieve and extract data about relevant studies. We identified 2248 citations, of which 21 studies met criteria for inclusion; 20 studies were randomized controlled trials, and 1 was a controlled clinical trial. The following information and communication technologies were evaluated: websites (5 studies), computer software (3 studies), Web-based workshops (2 studies), computerized decision support systems (2 studies), electronic educational game (1 study), email (2 studies), and multifaceted interventions that consisted of at least one information and communication technology component (6 studies).

**Results:**

Website studies demonstrated significant improvements in perceived usefulness and perceived ease of use, but not for knowledge, reducing barriers, and intention to use clinical practice guidelines. Computer software studies demonstrated significant improvements in perceived usefulness, but not for knowledge and skills. Web-based workshop and email studies demonstrated significant improvements in knowledge, perceived usefulness, and skills. An electronic educational game intervention demonstrated a significant improvement from baseline in knowledge after 12 and 24 weeks. Computerized decision support system studies demonstrated variable findings for improvement in skills. Multifaceted interventions demonstrated significant improvements in beliefs about capabilities, perceived usefulness, and intention to use clinical practice guidelines, but variable findings for improvements in skills. Most multifaceted studies demonstrated significant improvements in knowledge.

**Conclusions:**

The findings suggest that health professionals’ perceived usability and practice behavior change vary by type of information and communication technology. Heterogeneity and the paucity of properly conducted studies did not allow for a clear comparison between studies and a conclusion on the effectiveness of information and communication technologies as a knowledge translation strategy for the dissemination of clinical practice guidelines.

## Introduction

Success in regularly transferring research knowledge into clinical practice has been limited [1]. Evidence-based clinical practice guidelines (CPGs) are often not implemented effectively, resulting in the failure to achieve optimal health outcomes for patients [2]. Thus, efforts to reduce the knowledge-to-action gap remain a constant challenge among researchers and health professionals.

Knowledge translation (KT), the process of implementing knowledge into action, can provide methods for closing the knowledge-to-action gap [3]. With the emerging appeal of Web-based KT resources that allow for potential widespread reach through self-paced, self-directed learning, the Internet has become an important platform for KT initiatives such as CPG dissemination [4]. Information and communication technologies (ICTs) are defined as “technologies that provide access to information through telecommunications…[focusing] primarily on communication technologies. This includes the Internet, wireless networks, cell phones, and other communication mediums” [5]. ICTs have the potential to improve accessibility to CPGs. For example, digital CPGs can be continuously reviewed and updated with new evidence, while having the potential to be widely disseminated [6]. Furthermore, these Web-based tools provide both clinicians and consumers with a convenient method to access evidence-based CPGs [6].

Teaching modalities for medical education, including CPG dissemination, have evolved [7]. The development and implementation of novel teaching and dissemination strategies was prompted by research findings showing that traditional didactic seminars do not always modify behavior and learning competency [7]. Grimshaw et al [8] concluded that the evidence to guide choice of KT strategies targeting health professionals is incomplete. While the evidence of traditional KT strategies, such as printed educational materials [9], educational meetings [10], educational outreach [11], local opinion leaders [12], and audit and feedback [13], focusing on practice behavior change targeting health care professionals has been summarized [8], we have limited knowledge of the perceived usability and practice behavior among health professionals when using novel KT strategies such as ICTs for the dissemination of CPGs.

The objective of this systematic review was to summarize the evidence pertaining to the use of ICTs for the dissemination of CPGs to health professionals. Specifically, with this review we sought to provide new knowledge on health professionals’ perceived usability and change in practice behavior when using ICTs to disseminate CPGs.

## Methods

We conducted this systematic review using the Preferred Reporting Items for Systematic Reviews and Meta-Analyses (PRISMA) guidelines [14]. To summarize the evidence, we used a systematic approach to retrieve relevant articles from the literature. Articles were selected for this review using the following predefined selection criteria guided by the population, intervention, comparison, outcome, and study design (PICOS) process.

We excluded studies if they did not meet the selection criteria (Table 1). We also excluded duplicate publications, narrative reviews, case series, case reports, data presented in abstract form only, conference proceedings, study protocols, and publications not written in English.

**Table 1:** Study selection criteria.

Criterion	Definition
Population	Health professionals (eg, physicians including medical residents, nurses, and physiotherapists)
Intervention	Information and communication technologies for disseminating clinical practice guidelines
Comparator	Information and communication technologies compared with each other or control (eg, no intervention)
Outcomes	Usability (eg, perceived usefulness and perceived ease of use)
	Practice behavior (eg, barriers, knowledge, skills, social/professional role and identity, optimism, beliefs about capabilities, beliefs about consequences, intentions, memory/attention/decision, environmental context and resources, social influences, and emotion)
Study design	Randomized controlled trials
	Nonrandomized comparative controlled trials

The literature search was performed by an information specialist. Published literature was identified by searching the following bibliographic databases up to the end of December 2015: MEDLINE, Cochrane Central Register of Controlled Trials, EMBASE, CINAHL, ERIC, and PsycINFO. The search was performed using terms to identify peer reviewed research in which ICTs and CPG dissemination were important features (Multimedia Appendix 1). A search of gray literature (literature that is not commercially published) was conducted by searching Google and other Internet search engines for additional Web-based publications. In addition, the searches were supplemented by hand searching the bibliographies of key articles. To ensure all ICTs would be captured in the literature search, including those that are older and established (eg, email), we did not place any date limits.

**Figure 1:**
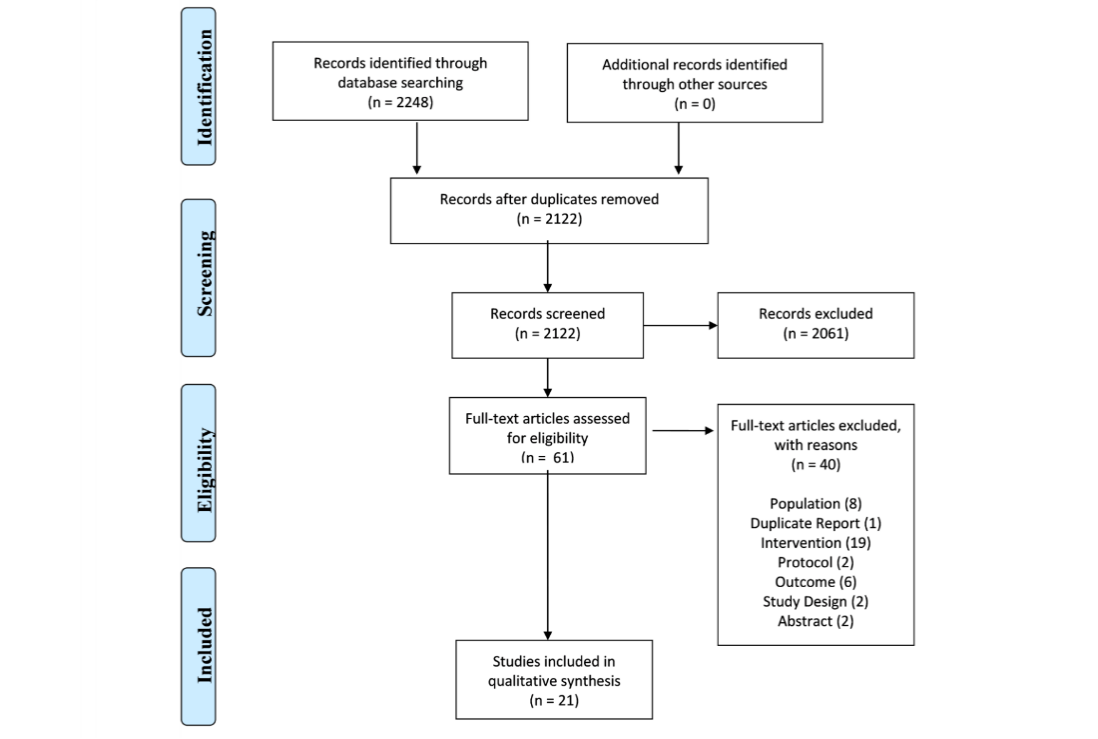
Preferred Reporting Items for Systematic Reviews and Meta-Analyses (PRISMA) flow diagram of included studies.

Titles and abstracts of all citations retrieved from the literature search were independently screened by 2 reviewers using Covidence (Veritas Health Innovation Ltd), a Web-based systematic review software. Full-text articles were then independently reviewed based on the selection criteria. Disagreements were resolved through discussion until consensus was reached. Figure 1 presents the study selection process in a PRISMA flow diagram.

Both descriptive data and results were extracted by 1 reviewer from each eligible article. The extraction was subsequently verified by a second reviewer. Data extraction forms were designed a priori to document and tabulate relevant study and patient characteristics, study findings, and authors’ conclusions. We did not use data from figures if the data were not explicit. Studies were categorized by the type of ICT intervention used.

One reviewer independently assessed the quality of each study using the Cochrane risk of bias tool [15], which was subsequently checked for accuracy by a second reviewer. Disagreements were resolved through consensus. Risk of bias was assessed at the study level.

Given the broad inclusion criteria and heterogeneity of the interventions and methodological characteristics of included studies (guided by PICOS), we deemed a meta-analysis to be inappropriate, and we therefore conducted a narrative synthesis and summary of study findings. The outcomes of interest were the usability of the ICT intervention and practice behavior change.

### Usability

The usability outcomes were guided by the technology acceptance model (TAM2) [16], which illustrates that behavior intention to use a system is determined by perceived usefulness and perceived ease of use. Perceived usefulness is defined by Venkatesh and Davis [16] as “the extent to which a person believes that using the system will enhance his/her job performance” (pg 187), and perceived ease of use is defined as “the extent to which a person believes that using the system will be free of effort” (pg 187).

### Practice Behavior

The theoretical domains framework (TDF) guided the practice behavior change outcomes [2]. The TDF identifies numerous behavior constructs and consists of 12 domains: (1) knowledge, (2) skills, (3) social or professional role and identity, (4) beliefs about capabilities, (5) beliefs about consequences, (6) motivation and goals, (7) memory, attention, and decision processes, (8) environmental context and resources, (9) social influences, (10) emotion regulation, (11) behavioral regulation, and (12) nature of the behavior. We categorized practice behavior outcomes by the domains listed above.

## Results

We identified a total of 2248 citations through the initial search. After removing duplicates, we screened 2122 publication abstracts and titles. We assessed the full texts of 61 articles; of these, we excluded 40 for the following reasons: irrelevant population (8 studies), duplicate report (1 study), irrelevant intervention (19 studies), study protocol (2 studies), irrelevant outcome (6 studies), inappropriate study design (2 studies), and presented as abstract only (2 studies). The excluded studies are listed in Multimedia Appendix 2. Figure 1 shows the PRISMA flow diagram.

Of the 21 studies that we included in our systematic review, 20 were randomized controlled trials (95%) and 1 was a controlled clinical trial (5%) [17-37] (Table 2). There were 7 primary ICT interventions that were used to disseminate CPGs: websites [17,22-25], computer software [26-28], Web-based workshops [20,29], computerized decision support systems (CDSSs) [30,31], electronic educational game [21], email [19,32], and multifaceted interventions that consisted of at least one ICT component [18,33-37].

**Table 2:** Type of information and communication technology (ICT) used in each included study.

ICT intervention	Number of studies	Studies
Website	5	Balamuth et al [22]; Bell et al [23]; Schroter et al [17]; Sassen et al [24]; Wolpin et al [25]
Computer software	3	Bullard et al [26]; Butzlaff et al [27]; Jousimaa et al [28]
Web-based workshops	2	Epstein et al [20]; Fordis et al [29]
Computerized decision support system	2	Gill et al [30]; Peremans et al [31]
Electronic educational game	1	Kerfoot et al [21]
Email	2	Lobach [19]; Stewart et al [32]
Multifaceted^a^	6	Bernhardsson et al [33]; Chan et al [34]; Desimone et al [35]; McDonald et al [36]; Fretheim et al [18]; Shenoy [37]

^a^Multifaceted intervention that consisted of at least one ICT component.

Multimedia Appendix 3 presents the study characteristics. Of the included studies, 11 (52%) involved only physicians [20-24,27-30,32,37], 3 (14%) involved only medicine residents and fellows (family or internal) [23,25,35], 3 (14%) involved only nurses [31,34,36], and 1 (5%) involved physiotherapists [33]. A total of 2 studies (10%) assessed both nurses and physicians [17,18], and another study (5%) assessed the combination of physicians, nurses, and medical residents [19].

In 8 studies, there was no comparison with an intervention [19,27,30,31,33,34], usual care [36], or usual education [35]. Another 2 studies were compared with a waiting list [24,32], 10 studies were compared with active interventions [17,18,21-23,25,26,28,29,37], and 1 study was a pre-post design where assessments were conducted before and after the ICT intervention [20]. In terms of location, 10 studies were conducted in the United States [19,20,22,23,25,29,30,35-37], 3 were in Canada [26,32,34], 7 were in Europe [17,18,24,27,28,31,33], and 1 was an international study conducted in 63 countries [21]. Study durations and follow-up ranged from immediate posttest to 1 year postintervention.

### Websites

The use of a website for the dissemination of CPGs to health professionals was assessed in 5 studies [17,22-25] (Table 3). Balamuth et al [22] compared a Web-based 1-page summary sheet of guidelines (n=128) with a weblink to guidelines (n=109) among physicians after 6 weeks. Schroter et al [17] compared an interactive Web-based tool combined with Web-based didactic material (n=527) with Web-based didactic material alone (n=527) among physicians and nurses after 4 months. Sassen et al [24] compared a website with educational modules (n=48) with a waiting list group (n=33) among orthopedic surgeons after 12 months. A further 2 studies involved only medicine residents and fellows [23,25]. Bell et al [23] compared self-study Web-based guidelines (n=79) with print-based guidelines (n=83) among family and internal medicine residents at immediate posttest and at 4 to 6 months postintervention. Wolpin et al [25] compared a website with enhanced learning modules (n=33) with a website containing usual care instructions (n=36) among medicine residents and fellows at 12 weeks postintervention.

**Table 3:** Summary of findings of included studies by primary information and communication technology (ICT) intervention.

ICT intervention	Study	Interventions	Outcome(s)	Effect size	Conclusion
**Website**					
	Balamuth, 2010 [22]	Web-based 1-page summary sheet of guidelines (n=128) Weblink to guidelines (n=109)	*Knowledge*: correctly diagnosed patients OR^a^ (95% CI)	0.82 (0.49-1.4)	No statically significant difference between 2 groups in correctly diagnosing patients according to guidelines. Participants using the Web-based 1-page summary reported that the supplemental materials were more simple to use when compared with the weblink group.
			*Perceived ease of use*: simplicity of supplemental materials OR (95% CI)	6.1 (2.8-13.6)	
	Bell, 2000 [23]	Self-study Web-based guidelines (n=79) Print-based guidelines (n=83)	*Knowledge*: median (95% CI) score (out of 20) after immediate posttest	Web-based: 15.0 (14.0-15.0) Print based: 14.5 (14.0-15.0) *P*=.20	No statistically significant difference in knowledge at immediate posttest or after 4-6 months. Web-based guideline users were more satisfied with learning.
			*Knowledge*: median (95% CI) score (out of 20) after 4-6 months	Web-based: 12.0 (11.0-13.0) Print based: 11.0 (10.0-12.0); *P*=.12)	
			*Perceived ease of use*: median (95% CI) learner satisfaction scores (range 5-20, higher = better)	Web-based: 17.0 (16.0-18.0) Print-based: 15.0 (15.0-16.0); *P*<.001	
	Schroter, 2011 [17]	Website with educational modules (n=48) Waiting list (n=33)	*Knowledge*: mean % change (SD) from baseline knowledge at 4 months	Web-based plus Web material: 47.4% (12.6) to 66.8% (11.5) Web-based material only: 47.3% (12.9) to 67.8% (10.8); *P*=.19	No statistically significant differences in knowledge change or usability between the 2 groups. Participants in Web-based tool plus Web material group found it to be useful. Usefulness was not measured in the other group.
			*Perceived usefulness*: % of participants who reported the tool to be very useful/useful	Web-based plus Web material: 77% Web-based material only: NR^b^	
	Sassen, 2014 [24]	Website with educational modules (n=48) Waiting list (n=33)	*Intention* to use material to educate patients: mean (SD) score out of 7 (higher = easier) at baseline and 12 months	Website: 6.25 (1.00), 6.06 (1.11) Waiting list: 5.87 (1.15), 6.02 (0.91), *P*=.12	No statistically significant differences in intention to use and barriers between interventions groups at 12 months.
			*Barriers* to using the material to educate patients: mean (SD) score out of 7 (higher = easier) at baseline and 12 months	Website: 3.11 (1.17), 3.18 (1.12) Waiting list: 2.78 (1.01), 2.63 (0.96), *P*=.46	
	Wolpin, 2011 [25]	Website enhanced learning (additional case studies) (n=33) Website with usual care instructions (same content, without case studies) (n=36)	*Knowledge*: mean (SD) score % on knowledge content of CPGs^c^ pretest and immediate posttest	Overall (pooled both groups): 79.28% (12.17), 82.32% (13.84), *P*=.10 Website (enhanced) 78.18% (11.1), 79.39% (15.0) Website (usual): 80.28% (13.2), 85.0% (12.3)	No statistically significant difference in knowledge or satisfaction at posttest between intervention groups. No statistically significant differences were seen between interventions groups for both outcomes.
			*Perceived ease of use*: overall satisfaction with learning experience, mean (SD) score (1-5, higher = very satisfied), pretest and immediate posttest	Overall (pooled both groups): 4.08 (0.860) Website (enhanced) 78.18 (11.1), 79.39 (15.0) Website (usual): 80.28 (13.2), 85.0 (12.3), *P*=.13	
**Computer software**					
	Bullard, 2004 [26]	Wirelessly networked mobile computer program (n=10)^d^ Desktop computer program (n=10)^d^	*Perceived usefulness*: “impact on efficiency” mean (95% CI) score out of 7	Wireless: 3.2 (2.6-3.8) Desktop: 4.3 (4.0-4.6), *P*=.02	Statistically significant greater satisfaction for several items (“impact on efficiency,” “increase use of CPGs,” and “saving time”) when using the wireless computer compared with the desktop computer. Other satisfaction items such as “configuration,” “availability,” “reduced communication with staff and patients,” and “accessibility” did not show statistically significant differences (results not shown). Participants appeared to be indifferent regarding the usability of the wireless computer for their efficiency.
			*Perceived usefulness*: “increased use of CPGs” mean (95% CI) score out of 7 (7 = excellent)	Wireless: 4.1 (3.6-4.6) Desktop: 3.5 (2.9-4.0), *P*=.03	
			*Perceived usefulness*: “wireless computer program made participant more efficient,” mean (95% CI) score out of 7 (7 = strongly agree)	Wireless: 3.30 (2.33-4.27) Desktop: NR	
	Butzlaff, 2004 [27]	CPGs via CD-ROM/Internet (n=53) No intervention (n=66)	*Knowledge*: median (IQR^e^) score out of 25 at baseline	CD/Internet: 13 (12-16) No intervention: 13 (10-15.25), *P*=.40	There was no statistically significant difference between intervention groups at baseline and ~70 postintervention in knowledge scores.
			*Knowledge*: median (IQR) score out of 25 at ~70 days posttest	CD/Internet: 15 (12-17) No intervention: 13 (11-15.25), *P*=.10	
	Jousimaa, 2002 [28]	CD-ROM computer-based guidelines (n=72) Textbook-based guidelines (n=67)	*Skills*: compliance with CPGs, “laboratory examinations,” OR (95% CI)	1.07 (0.79-1.44)	There was no statistically significant difference between intervention groups for compliance with CPGs for laboratory, radiological, or physical examinations.
			*Skills*: compliance with CPGs, “radiological examinations,” OR (95% CI)	1.09 (0.81-1.46)	
			*Skills*: compliance with CPGs, “physical examinations,” OR (95% CI)	0.74 (0.51-1.06)	
				
				
				
				
				
				
				
				
				
				
**Web-based workshops**					
	Epstein, 2011 [20]	Web-based didactic education session/workshop (n=27) No intervention (received intervention after 6 months) (n=22)	*Skills*: compliance with CPGs, “use of parent ratings of ADHD^[f]^ during assessment,” mean % change from baseline at 6 months	Web: 23.8% No intervention: 5.7%, *P*=.03	Statistically significant changes from baseline to 6 months were seen among participants complying with CPG-recommended ADHD care practices, with the exception of 1 recommendation, “Use of parent ratings of ADHD to monitor treatment responses” (results not shown).
			*Skills*: compliance with CPGs, “use of teacher ratings of ADHD during assessment,” mean % change from baseline at 6 months	Web: 22.6% No intervention: 6.0%, *P*=.04	
			*Skills*: compliance with CPGs, “use of [*Diagnostic and Statistical Manual of Mental Disorders* (Fourth Edition)] ADHD criteria during assessment,” mean % change from baseline at 6 months	Web: 47.3% No intervention: 17.9%, *P*=.03	
			*Skills*: compliance with CPGs, “use of outside provider for ADHD diagnosis,” mean % change from baseline at 6 months	Web: –60.7% No intervention: –10.7%, *P*<.001	
			*Skills*: compliance with CPGs, “use of teacher ratings of ADHD to monitor treatment responses,” mean % change from baseline at 6 months	Web: 38.7% No intervention: 6.3%, *P*=.003	
	Fordis, 2005 [29]	Live Web-based CME^g^ workshop (n=51) Web-based CME workshop (n=52) No intervention (n=20)	*Knowledge*: the 2 active CME interventions combined: mean % change (95% CI) from baseline to immediate posttest	31.0% (95% CI 27.0%-35.0%), *P*<.001	A statistically significant improvement in knowledge was seen over time for both Web-based interventions groups. A statistically significant decrease in appropriately screening patients was seen in the live Web-based CME group at 12 weeks posttest compared with baseline. No statistically significant differences were seen for screening patients between interventions groups. There was a statistically significant increase in the proportion of patients appropriately treated by the Web-based CME group compared with the live CME and control groups. Participants in the Web-based interventions were satisfied with the learning experience.
			*Knowledge*: the 2 active CME interventions combined: mean % change (95% CI) from baseline to 12 weeks posttest	36.4% (95% CI 32.2%-40.6%), *P*<.001	
			*Knowledge*: the 2 active CME interventions combined: mean % change (95 CI) from immediate posttest to 12 weeks posttest	5.4% (95% CI 2.6%-8.2%)	
			*Skills:* patients appropriately screened for dyslipidemia, mean % change (95% CI) from baseline to 12 weeks postintervention	Live Web-based: −3.3 (−5.9 to −0.7) Web-based: −0.1 (−2.9 to 2.6) No intervention: −0.8 (−3.5 to 1.8), *P*=.24	
			*Skills*: patients appropriately treated for dyslipidemia, mean % change from baseline to 12 weeks postintervention	Live Web-based: −1.1 (−4.9 to 2.7) Web-based: 5.0 (1.0-9.1) No intervention: 1.2 (−2.8 to 5.1), *P*=.04	
			*Perceived usefulness*: % of participants satisfied with the learning experience	Live Web-based: 100% (49/49) Web-based: 94% (44/47) No intervention: NR	
**Computerized decision support system**					
	Gill, 2011 [30]	EHR^h^-based clinical decision support (n=53) No intervention (n=66)	*Skills*: % of patients receiving guideline-concordant care, OR (95% CI)	EHR: 25.4% No intervention: 22.4%, OR 1.19 (1.01-1.42)	There was a statistically significant difference favoring the EHR intervention compared with no intervention for the proportion of patients receiving guideline-concordant care.
	Peremans, 2010 [31]	EHR-based clinical decision support (n=15) Empowered patient group (n=15) No intervention (n=13)	*Skills*: consultation and prescribing skills based on a 48-item checklist, mean difference (95% CI) from baseline to 5 months postintervention	EHR: –1.79 (–4.97 to 1.65) Empowered: 4.92 (1.96-7.89) No intervention: –0.91 (–3.37 to 1.92)	The empowered patient group was the only group that had improved consultation and prescribing skills scores after 5 months postintervention and the only intervention that demonstrated a statistically significant difference compared with no intervention.
**Electronic educations game**					
	Kerfoot, 2009 [21]	Electronic game/survey 2 questions every 2 days (n=735) Electronic game/survey 4 questions every 4 days (n=735)	*Knowledge*: median % (IQR) scores for knowledge test baseline	Electronic game 2 questions every 2 days: 48% (18) Electronic game 4 questions every 4 days: 45% (15)	Both electronic game cohorts demonstrated statistically significant improvements in knowledge compared with baseline.
			*Knowledge*: median % (IQR) scores for knowledge test postintervention (12 or 24 weeks), *P* value	Electronic game 2 questions every 2 days: 100% (3) Electronic game 4 questions every 4 days: 98% (8), *P*<.001	
				
				
				
				
				
				
				
				
				
				
**Email**					
	Lobach, 1996 [19]	Biweekly emails of computer-based audit/feedback program (n=22) No intervention (n=23)	*Skills*: median % (IQR) participant compliance with guidelines, *P* value	Email: 35.3% (NR^i^) No intervention: 6.1% (NR^i^), *P*=.01	The email intervention demonstrated statistical significance in greater compliance with guidelines compared with no intervention.
	Stewart, 2005 [32]	Email Web-based learning for 2 evidence-based modules (type 2 diabetes, prevention) (n=27) Waiting list (n=31)	*Knowledge*: mean (SD) score (out of 100) at baseline	Email (diabetes): 66.8 (14.1) Email (prevention): 53.8 (12.8) Waiting list (diabetes): 68.6 (10.4) Waiting list (prevention): 51.9 (9.5)	The intervention group (prevention module) demonstrated statistically significant improvements compared with the control group for knowledge at 2 and 6 months, as well as compliance at 6 months. There was no statistically significant difference with the diabetes modules.
			*Knowledge*: mean (SD) score (out of 100) at 2 months postintervention, *P* value	Email (diabetes): 72.7 (14.1) Email (prevention): 63.8 (17.6) Waiting list (diabetes): 67.7 (16.8), *P*=.57 Waiting list (prevention): 50.5 (13.8), *P*=.002	
			*Knowledge*: mean (SD) score (out of 100) at 6 months postintervention, *P* value	Email (diabetes): 73.2 (7.7) Email (prevention): 65.7 (15.2) Waiting list (diabetes): 68.6 (11.4), *P*=.14 Waiting list (prevention): 53.3 (10.5), *P*=.004	
			*Skills*: mean (SD) score for compliance with guidelines (out of 100) at baseline	Email (diabetes): 53.8 (12.5) Email (prevention): 52.2 (11.1) Waiting list (diabetes): 51.2 (11.6) Waiting list (prevention): 51.1 (14.4)	
			*Skills*: mean (SD) score for compliance with guidelines (out of 100) at 2 months postintervention, *P* value	Email (diabetes): 51.7 (12.9) Email (prevention): 52.2 (11.7) Waiting list (diabetes): 51.6 (9.5), *P*=.90 Waiting list (prevention): 47.7 (13.8), *P*=.11	
			*Skills*: mean (SD) score for compliance with guidelines (out of 100) at 6 months postintervention, *P* value	Email (diabetes): 47.1 (9.2) Email (prevention): 55.0 (10.0) Waiting list (diabetes): 50.8 (9.1), *P*=.14 Waiting list (prevention): 50.0 (14.4), *P*=.03	
				
				
				
**Multifaceted**					
	Bernhardsson, 2014 [33]	Multifaceted: implementation seminar/group discussion, website, and email reminders (n=168) No intervention (n=88)	*Knowledge*: change in % of participants who were aware that guidelines exist from baseline to 1-year follow-up, *P* value	Intervention: 27.9% No intervention: 7.3%, *P*=.02	There was a statistically significant difference favoring the intervention group for change in awareness, knowledge of where to find guidelines, and accessibility of guidelines at 1-year follow-up. There were no significant differences in frequent use of CPGs.
			*Knowledge*: change in % of participants who knew where to find guidelines from baseline to 1-year follow-up, *P* value	Intervention: 25.2% No intervention: 4.8%, *P*=.007	
			*Perceived ease of use*: change in % of participants who felt guidelines were easy to access from baseline to 1-year follow-up, *P* value	Intervention: 17.4% No intervention: −4.3%, *P*<.001	
			*Skills*: change in % compliance with use of CPGs (frequently or almost always)	Intervention: 9.2% No intervention: −0.2%, *P*=.30	
	Chan, 2013 [34]	Multifaceted: in-person education session and Web-based support (n=31) No intervention (n=22)	*Beliefs about capabilities*: change in % (95% CI) of participants who were self-confident in following CPGs at 2 weeks postintervention	Intervention: 25.9% (4.2 to 45.5) No intervention: 6.3% (−2.0 to 32.1)	There were statistically significant improvements in self-confidence to use, satisfaction in following, and willingness to follow CPGs among the intervention group at 2 weeks postintervention. There were no significant improvements among the control group.
			*Perceived usefulness*: change in % (95% CI) of participants who were satisfied in following CPGs at 2 weeks postintervention	Intervention: 40.7% (16.1-59.6) No intervention: −12.5 (−37.3 to 12.7)	
			*Intention*: willingness to use new CPGs, mean score change (95% CI) (out of 4, 4=all CPGs) at 2 weeks postintervention	Intervention: 0.74 (0.36-1.1) No intervention: 0.19 (−0.10 to 0.48)	
	Desimone, 2012 [35]	Multifaceted: in-person education, Web-based support, printed materials (n=11) Usual education (n=11)	*Knowledge*: mean % (SD) of correct responses (11 items) at baseline	Multifaceted: 69% (1.7) Usual education: 76% (1.2)	There was a statistically significant improvement in knowledge in both groups at 1 month postintervention. There were no observable differences between groups (between-group statistical analyses not performed).
			*Knowledge*: mean % (SD) of correct responses (11 items) at 1 month postintervention, *P* value	Multifaceted: 83% (2.1), *P*=.003 Usual education: 84% (1.4), *P*=.02	
	McDonald, 2005 [36]	Multifaceted: email reminder with provider prompts, patient education material, and clinical nurse specialist outreach (n=97) Email reminder of recommendations (n=121) Usual care (n=118)	*Skills*: adjusted mean difference in probability that participant assessed bowel movement based on CPG compared with usual care, *P* value	Email reminder: –5.7, *P*=.02 Multifaceted: –2.7, *P*=.26	In the email reminder intervention group, there was a decrease in performance, as the probability of nurses completing bowel movement assessments was statistically significantly lower compared with usual care. There was no statistically significant difference compared with the multifaceted group. Other nurse assessment and instruction practices did not reach statistical significance when the email reminder and multifaceted interventions were compared with usual care (results not shown).
	Fretheim, 2006 [18]	Multifaceted: educational outreach visit, audit and feedback at outreach visit, computerized reminders, risk assessment tools, patient information material, telephone follow-up (n=257) Passive guideline dissemination (no additional active promotion or encouragement for use of guidelines) (n=244)	*Skills*: mean change in % participants prescribing in concordance to CPGs from baseline to 12 months, between-group difference RR^j^ (95% CI)	Multifaceted: 11.5% Passive dissemination: 2.2%, 1.94 (1.49-2.49)	There was a statistically significant difference in participants prescribing in concordance to CPGs from baseline to 12 months favoring the multifaceted group compared with passive guidelines dissemination. No statistically significant differences were demonstrated for differences in participants performing risk assessments at 12 months.
			*Skills*: between-group difference in mean % participants performing risk assessments according to CPGs at 12 months, RR (95% CI)	1.04 (0.60-1.71)	
	Shenoy, 2013 [37]	Multifaceted: Web-based education, audit, feedback (n=24) Mailed guidelines (n=21)	*Knowledge*: mean change (95% CI) in total score (18 clinical vignettes) from baseline to 12 weeks postintervention	0.04 (1.22-1.31)	There was no statistically significant change in knowledge between intervention groups from baseline to 12 weeks postintervention. There was no statistically significant difference between intervention groups for the proportion of patients receiving CPG-adherent care at 12 weeks postintervention (results not shown).

^a^OR: odds ratio.

^b^NR: not reported.

^c^CPG: clinical practice guideline.

^d^Crossover design with same participants in both groups.

^e^IQR: interquartile range (25th to 75th percentile).

^f^ADHD: attention-deficit/hyperactivity disorder.

^g^CME: continuing medical education.

^h^EHR: electronic health record.

^i^IQR values illustrated in a diagram; however, values are not explicit.

^j^RR: relative risk.

#### Usability

Perceived usefulness was assessed in 1 study [17]. There was no statistically significant difference between intervention groups in regard to the proportion of physicians and nurses finding the intervention to be usable for integrating the learning into clinical practice. However, 76.7% (218/284) of physicians and nurses in the interactive Web-based tool plus Web-based didactic material found the intervention to be “very useful/useful.” Usability was not measured in the Web-based didactic material-alone group and no comparative statistical analyses were performed.

Perceived ease of use was assessed in 3 studies [22,23,25]. Balamuth et al [22] found that physicians using the Web-based 1-page summary reported that the supplemental materials were “simpler” to use than did the group using a weblink to guidelines (odds ratio, OR 6.1, 95% CI 2.8-13.6). In 1 of the studies involving only medicine residents and fellows by Bell et al [23], the median (95% CI) learner satisfaction scale score (out of 20) was statistically significantly greater (*P*<.001) in the self-study Web-based guidelines group (OR 17.0, 95% CI 16.0-18.0) than in the print-based guidelines group (OR 15.0, 95% CI 15.0-16.0). In Wolpin et al [25], the other study involving only medicine residents and fellows, there was no statistically significant difference in overall satisfaction with learning experience between the intervention groups.

#### Practice Behavior

Knowledge was assessed in 4 studies [17,22,23,25]. In all 4 studies, there was no statistically significant improvement in knowledge when compared with respective comparators.

Intention to use CPGs and reduction in barriers were assessed in 1 study [24]. There was no statistically significant difference between groups for intention to use material to educate patients, and no statistically significant difference in reduced barriers to using the material to educate patients.

### Computer Software

The use of computer software for the dissemination of CPGs among health professionals was assessed in 3 studies [26-28] (Table 3). Bullard et al [26] used a crossover design to compare a wirelessly networked mobile computer program with a desktop computer program among physicians (n=10) after 8-hour shifts. Butzlaff et al [27] compared CPGs provided by CD-ROM and Internet (n=53) with no intervention (n=66) among physicians after approximately 70 days. Jousimaa et al [28] compared CD-ROM computer-based guidelines (n=72) with textbook-based guidelines (n=67) among physicians after 1 month.

#### Usability

Perceived usefulness was assessed in 1 study [26]. Statistically significant mean (95% CI) satisfaction scores (out of 7, with 7 representing excellent) favored the wireless network mobile computer program group compared with the desktop computer program group for several items such as “impact on efficiency” (OR 3.2, 95% CI 2.6-3.8 vs OR 4.3, 95% CI 4.0-4.6, *P*=.02), “increased use of CPGs” (OR 4.1, 95% CI 3.6-4.6 vs OR 3.5, 95% CI 2.9-4.0, *P*=.03), and “saving time” (OR 3.1, 95% CI 2.3-3.9 vs OR 4.2, 95% CI 3.6-4.7, *P*=.05). Other satisfaction items such as “configuration,” “availability,” “reduced communication with staff and patients,” and “accessibility” did not show statistically significant differences between intervention groups. Physicians appeared to be indifferent regarding the usability of the wireless computer with respect to their efficiency, with a mean (95% CI) score (out of 7, with 7 representing strongly agree) of 3.30 (2.33-4.27). Usability of the desktop computer program was not assessed.

#### Practice Behavior

Knowledge was assessed in 1 study [27]. There was no statistically significant difference in knowledge scores between intervention groups.

Skills were assessed in 1 study [28]. There was no statistically significant difference between intervention groups for compliance skills with CPGs for laboratory, radiological, or physical examinations.

### Web-Based Workshops

The use of Web-based workshops for the dissemination of CPGs among health professionals was assessed in 2 studies [20,29] (Table 3). Epstein et al [20] compared a Web-based didactic education session or workshop (n=27) with no intervention (n=22) among pediatricians after 6 months. Participants in the Web-based didactic education workshop group received four 1-hour training sessions with instructions to use an Internet portal to assess attention-deficit/hyperactivity disorder (ADHD), titrate and monitor responses to medications, and communicate with patients and their parents and teachers using a Web-based report card. Fordis et al [29] compared a live Web-based continuing medical education (CME) workshop (n=51) with a Web-based (nonlive) CME workshop (n=52) and with no intervention (n=20) among physicians after 12 weeks.

#### Usability

Perceived usefulness was assessed in 1 study [29]. The proportion of physicians satisfied with the learning experience was 100% (49/49) for the live CME group and 94% (44/47) for the Web-based CME group. No comparative statistical analyses were performed for the perceived usefulness outcome.

#### Practice Behavior

Skills were assessed in both studies [20,29]. In Epstein et al [20], the Web-based didactic education workshop group demonstrated statistically significant improvements (mean percentage change from baseline) in ADHD care practices when compared with no intervention for the following CPG recommendations: “use of parent ratings of ADHD during assessment” (23.8% vs 5.7%, *P*=.03), “use of teacher ratings of ADHD during assessment” (22.6% vs 6.0%, *P*=.04), “use of DSM-IV [*Diagnostic and Statistical Manual of Mental Disorders* (Fourth Edition)] ADHD criteria during assessment” (47.3% vs 17.9%, *P*=.03), “use of outside provider for ADHD diagnosis” (–60.7% vs –10.7%, *P*<.001), and “use of teacher ratings of ADHD to monitor treatment responses” (38.7% vs 6.3%, *P*=.003). In Fordis et al [29], among the 3 intervention groups, there was no change from baseline screening levels following the intervention and no statistically significant differences between interventions groups. There was a statistically significant (*P*=.04) increase in the mean proportion (95% CI) of patients appropriately treated by the Web-based CME group (5.0%, 1.0%-9.1%) when compared with the live CME (−1.1%, −4.9% to 2.7%) and control groups (1.2%, −2.8% to 5.1%).

Knowledge was assessed in 1 study [29]. There was a statistically significant (*P*<.001) improvement in knowledge for both Web-based interventions groups combined, with a mean (95% CI) change of 31.0% (27.0%-35.0%) from baseline to immediate posttest, and 36.4% (32.2%-40.6%) to 12 weeks posttest.

### Computer Decision Support System

The use of CDSSs for the dissemination of CPGs among health professionals was assessed in 2 studies [30,31] (Table 3). According to Peremans et al [31], a CDSS is defined as “any software designed to directly aid clinical decision making, whereby individual patient records are matched with a computer database of guidelines” (pg 281). Peremans et al [31] compared an electronic health record (EHR)-based CDSS intervention (n=15) with a group receiving a visit by a simulated “empowered” patient (n=15) and with no intervention (n=13). Gill et al [30] compared an EHR-based CDSS intervention (n=53) with no intervention (n=66) among physicians and clinicians in ambulatory practices after 12 months.

#### Usability

Usability was not assessed in any of the included studies that used CDSSs for the dissemination of CPGs.

#### Practice Behavior

Skills were assessed in both studies [30,31]. In Peremans et al [31], the role of the simulated patient was to ask the physician specific clinical questions (a clinical scenario that was agreed upon by a panel of authors and researchers) regarding the prescribed pills she had received. The empowered-patient group was the only group that had statistically significant improved mean scores (out of 48 points) for consultation and prescribing skills after 5 months postintervention when compared with no intervention, with a mean (95% CI) difference of 4.92 (1.96-7.89). In Gill et al [30], there was a statistically significant difference favoring the EHR-based CDSS intervention compared with no intervention for delivering guideline-concordant care (OR 1.19, 95% CI 1.01-1.42).

### Electronic Educational Game

The use of an electronic educational game for the dissemination of CPGs among health professionals was assessed in 1 study [21] (Table 3). Kerfoot et al [21] compared an electronic educational game with a survey containing 2 questions distributed every 2 days (n=735) with a group receiving the same game, but with a survey containing 4 questions distributed every 4 days (n=735) among urologists after 34 weeks.

#### Usability

Usability was not assessed in Kerfoot et al [21].

#### Practice Behavior

Both game groups demonstrated statistically significant (*P*<.001) improvements in knowledge compared with baseline, with median scores of 48.0% (interquartile range, IQR 18) versus 100.0% (IQR 3) for the electronic game cohort answering 2 questions every 2 days, and 45.0% (IQR 15) versus 98.0% (IQR 8) for the cohort answering 4 questions every 4 days.

### Email

The use of email for the dissemination of CPGs among health professionals was assessed in 2 studies [19,32] (Table 3). Lobach [19] compared biweekly emails of a computer-based audit and feedback program (n=22) with no intervention (n=23) among physicians, general internists, nurses, physician assistants, and family medicine residents after 12 weeks. Stewart et al [32] examined the use of email to disseminate 2 separate evidence-based modules on diabetes and prevention (n=27) compared with a waiting list (n=31) among physicians after 6 months.

#### Usability

Usability was not assessed in any of the included studies that used email for the dissemination of CPGs.

#### Practice Behavior

Skills were assessed in both studies [19,32]. In Lobach [19], there was a statistically significant difference favoring the email intervention compared with no intervention for median rate of compliance with CPGs (35.3% vs 6.1%, *P*=.01). In Stewart et al [32], there was a statistically significant difference (*P*=.03) in skills favoring the email intervention compared with the waiting list, with mean (SD) compliance scores (out of 100) of 55.0 (10.0) versus 50.0 (14.4) for the prevention modules at 6 months. There was no statistically significant difference in compliance scores between intervention groups for the diabetes modules at 6 months and for both modules at 2 months.

Knowledge was assessed in 1 study [32]. There was a statistically significant difference (*P*=.002) favoring the email intervention compared with the waiting list, with mean (SD) knowledge scores (out of 100) of 63.8 (17.6) versus 50.5 (13.8), and 65.7 (15.2) versus 53.3 (10.5) for the prevention modules at 2 months and 6 months, respectively. There was no statistically significant difference in knowledge scores between intervention groups for the diabetes modules at 2 and 6 months.

### Multifaceted ICT Interventions

The use of a multifaceted intervention including an ICT with more than one CPG dissemination strategy among health professionals was assessed in 6 studies [18,33-37] (Table 3). Bernhardsson et al [33] compared the combination of an implementation seminar with group discussion, a website, and email with no intervention (n=88) among physiotherapists after 12 months. Shenoy [37] compared the combination of Web-based education and audit and feedback (n=24) with mailed CPGs (n=21) among physicians after 5 months. Fretheim et al [18] compared the combination of an educational outreach visit, audit and feedback at the outreach visit, computerized reminders, risk assessment tools, patient information material, and telephone follow-up (n=257) with passive guideline dissemination (no additional active promotion or encouragement for the use of guidelines) (n=244) among physicians and practice nurses after 45 days. Chan et al [34] compared the combination of an in-person education session and Web-based support (n=31) with no intervention (n=22) among nurses after 2 weeks. Desimone et al [35] compared the combination of in-person education, Web-based support, and printed materials (n=11) with usual education (n=11) among internal medicine residents after 4 weeks. McDonald et al [36] compared the combination of email reminders with provider prompts, patient education material, and clinical nurse specialist outreach (n=97) with email reminders of recommendations only (n=121) and usual care (n=118) among primary care and family medicine residents after 24 months.

#### Usability

Usability was assessed in 1 study [33]. There was no statistically significant difference between intervention groups for the change in proportion of physiotherapists who felt the CPGs were easy to access and the proportion of those who used the CPGs frequently.

Perceived usefulness was assessed in 1 study [34]. There was a statistically significant improvement in the proportion of nurses who were satisfied in following the CPGs at 2 weeks postintervention compared with baseline among the multifaceted intervention group, with a mean (95% CI) of 40.7% (16.1%-59.6%).

#### Practice Behavior

Knowledge was assessed in 3 studies [33,35,37]. In Bernhardsson et al [33], there were statistically significant improvements from baseline favoring the intervention group compared with no intervention for the proportion of physiotherapists who were aware that guidelines exist (27.9% vs 7.3%, *P*=.02) and the proportion of physiotherapists who were aware of where to find guidelines (25.2% vs 4.8%, *P*=.007). In Shenoy [37], there was no statistically significant improvement in knowledge among either the multifaceted intervention or the mailed guidelines groups. In the study involving only medicine residents and fellows by Desimone et al [35], there was a statistically significant improvement in correct responses (out of 11 items) from baseline in both intervention groups, with mean (SD) proportions for the multifaceted intervention group (83%, SD 2.1% vs 69%, SD 1.7%, *P*=.003) and the usual education group (84%, SD 1.4% vs 76%, SD 1.2%, *P*=.02).

Skills were assessed in 3 studies [18,33,36]. In McDonald et al [36], the probability of nurses completing bowel movement assessments was statistically significantly lower in the email reminder intervention group (*P*=.02) than in the usual care group, with an adjusted mean difference of –5.7% (89.0% vs 94.7%), representing a decrease in performance. There was no statistically significant difference compared with the multifaceted intervention group. Other nurse assessment and instruction practices did not reach statistical significance when the email reminder and multifaceted interventions were compared with usual care. In Fretheim et al [18], there was a statistically significant difference in the proportion of physicians and practice nurses prescribing in concordance to CPGs from baseline to 12 months favoring the multifaceted group (11.5%) compared with the passive guidelines dissemination group (2.2%), with a relative risk (95% CI) of 1.94 (1.49-2.49). There was no statistically significant difference between intervention groups for physicians and practice nurses performing risk assessments at 12 months. In Bernhardsson et al [33], there was no statistically significant difference between intervention groups for change in the proportion of physiotherapists who “frequently or almost always” used the CPGs.

Beliefs about capabilities and intention to use CPGs were assessed in 1 study [34]. There was a statistically significant improvement in the proportion of nurses who were self-confident in following the CPGs at 2 weeks postintervention compared with baseline among the multifaceted intervention group, with a mean (95% CI) of 25.9% (4.2%-45.5%). There was a statistically significant improvement in intention to use the new CPGs when compared with baseline among the multifaceted intervention group, with a mean (95% CI) change in score (out of 4, with 4 representing willingness to use all CPGs) of 0.74 (0.36-1.1). There was no statistically significant improvement among the control group for each of the outcomes listed above.

## Discussion

The aim of this review was to identify research on health professionals’ perceived usability and practice behavior with ICTs for the dissemination of CPGs. In summary, results varied by the type of ICT used. While rapidly changing technologies may pose challenges for the development, implementation, and evaluation of ICT-based interventions, as they may be associated with greater barriers for adoption by health professionals [38], there were no apparent trends when comparing established and older ICTs (eg, email and computer software) versus newer emerging ICT interventions (eg, electronic educational games, Web-based workshops, and the multifaceted ICT interventions). Studies using websites to disseminate CPGs [17,22-25] demonstrated no improvements in knowledge [17,22,23,25], reduced barriers [25], or intentions to use CPGs [25]. There were positive effects for perceived usefulness [17] and perceived ease of use [22,23] (2 of 3 studies). Studies using computer software [26-28] demonstrated no improvements in knowledge [27] or skills [28], but an effect on perceived usefulness [26]. We found that 2 studies using Web-based workshops [20,29] demonstrated improvements in knowledge [29] and perceived usefulness [29] and skills [20,29]. Studies using CDSSs demonstrated variable results for skills, as 1 study [30] demonstrated a positive effect, while the other did not [31]. While both studies were compared with no intervention, it should be noted that in the latter study [31], the non-ICT intervention (empowered patient group) was the only group that demonstrated a positive effect when compared with no intervention. The 1 study that used an electronic educational game [21] demonstrated an improvement in knowledge. Studies using email [19,32] demonstrated improvements in knowledge [32] and skills [19,32]. Studies using multifaceted ICT interventions [18,33-37] demonstrated improvements in knowledge [33,35] (2 of 3 studies), perceived usefulness [34], perceived ease of use [33], intention to use CPGs [34], beliefs about capabilities [33], and skills [37] (1 of 2 studies). While the multifaceted interventions in this review mostly demonstrated positive findings for improvements in usability and practice behavior, it remains unclear whether they are in fact superior to single interventions. Grimshaw et al [8] revealed that effect sizes in multifaceted interventions do not necessarily increase with increasing number of components, and these types of interventions appear to be more costly than single interventions. Similarly, a review by Squires et al [39] concluded that there is a lack of compelling evidence to demonstrate that multifaceted interventions are more effective than single interventions.

Outcome selection was guided by both the TAM2 [16] and the TDF [2]. We chose the TAM2 because it was originally designed to predict ICT acceptance and usage in the workplace and has been widely used for diverse sets of ICT users [40]; we chose the TDF because it simplifies and integrates many behavior change theories, including social cognitive theory, learning theory, and diffusion theory [2]. The TAM2 is a validated and robust theoretical framework that has been used for predicting and explaining behavior related to ICTs [16]. In addition to cognitive instrumental processes, the TAM2 encompasses social influence processes, including subjective norms, which have shown to explain the perceived usefulness of ICTs [41]. Developed from a synthesis of psychological theories, the TDF is an integrative framework that has been shown to be useful and flexible for the assessment of behavior change and barriers among a diverse group of health professionals working in various clinical settings [42]. Together, both theoretical frameworks provided a comprehensive list of outcomes to measure health professionals’ usability and practice behavior change of ICTs for the dissemination of CPGs.

The variable findings in knowledge improvement are supported by a recent systematic review [7] of educational strategies for teaching medical trainees, which found no difference in learner outcomes when comparing lecture-based versus Web-based strategies. While previous reviews have assessed interventions for promoting ICT adoption [43] and KT dissemination strategies focusing on practice behavior change among health professionals [8] distinctly, this systematic review adds to the body of literature by summarizing current evidence pertaining to health professionals’ perceived usability and practice behavior change with ICTs, specifically for the dissemination of CPGs. A systematic review by Gagnon et al [43] concluded that there is very limited evidence on effective interventions promoting the adoption of ICTs by health care professionals, while a systematic review by Grimshaw et al [8] concluded that the evidence to guide the choice of KT strategies targeting health professionals is incomplete. Understanding how health professionals engage with and use ICTs to access CPGs will enable health care provider organizations to create content that is more Web friendly [44]. While the evidence is limited, studies of ICTs included in this review have shown promising findings. ICTs are novel ways of disseminating CPGs, compared with more traditional methods such as printed educational materials [9], educational meetings [10], educational outreach [11], local opinion leaders [12], and audit and feedback [13]. This review highlights which ICTs have been successfully used as a dissemination strategy for CPGs; however, it remains unclear whether one ICT is more effective than another. It is also unclear whether other ICTs not captured in this review, such as social media, can be used as effective dissemination strategies for CPGs. Further research, by conducting well-designed randomized controlled trials, is necessary to determine whether the use of ICTs is an effective strategy to disseminate evidence-based medicine to health professionals. There were differences in study durations and measurements among the included studies. As none of the studies measured sustainability, researchers should consider what is an appropriate time frame to expect meaningful differences in behavior change. Future studies, designed to compare these strategies head-to-head, would provide further guidance. While the scope of the review focused on the dissemination of CPGs to health professionals, future research should also assess how ICT dissemination strategies can be used as a tool to share information between health professionals and patients. As only 1 of the included studies [24] assessed barriers, future research should consider barriers as a crucial outcome of interest.

### Strengths and Limitations

The strengths of this systematic review include the broad eligibility criteria that we used, allowing for numerous types of ICTs and various health professional populations (ie, physicians including medical residents, nurses, and physiotherapists) to be included and summarized in this review. Additionally, we used a systematic approach to review the literature and assessed the methodological quality of each included study. This systematic review was conducted following the PRISMA checklist [14].

Nevertheless, there are limitations of this review that should be considered. We did not include information published in languages other than English; thus, we may have excluded some relevant findings. The small number of included studies per ICT and the heterogeneity between studies in regard to the included health professional populations, definitions of outcomes assessed, selected comparators (some compared interventions against no intervention, while others used active comparators), and duration of studies did not allow for comparisons between studies. As a result, we were not able to calculate pooled effect sizes or perform meta-analyses. The terminology of outcomes in the included studies sometimes differed from the identified concepts in the TAM2 and domains of the TDF that we used to define the usability and practice behavior change outcomes, respectively. Several studies measured numerous outcomes, and it remains uncertain whether these studies were adequately powered to detect meaningful differences. Furthermore, the overall findings were limited by the high loss to follow-up in numerous studies [17,21,23,25,30,32,34,36]. While reasons for loss to follow-up remain unclear, one potential cause as suggested by study authors may be professional or organizational barriers related to the use of these ICTs. CPG dissemination and KT strategies should be tailored and driven by barriers to improve adherence in practice [44].

The authors of the included studies did not always assess the quality of information being presented or quality of ICT. The quality of information being presented was previously assessed and deemed appropriate by authors in 4 of 5 (80%) studies using websites [17,23-25], 1 of the 2 (50%) studies using Web-based workshops [29], the study using an electronic educational game [21], 1 of 3 (33%) studies using computer software [26], both studies using email [19,32], both studies using CDSSs [30,31], and 4 of 6 studies (67%) using a multifaceted intervention including an ICT [33,35-37]. It was unclear whether the quality of information was assessed and deemed appropriate in the remaining studies. The quality of the ICT was assessed and deemed appropriate in 2 of 5 studies (40%) using websites [24,25], 1 of the 2 (50%) studies using Web-based workshops [29], the study using an electronic educational game [21], 1 of 3 (33%) studies using computer software [26], 1 of 2 (50%) studies using email [19], and 1 of 6 studies [35] using a multifaceted intervention including an ICT. In studies using CDSSs, the quality of the ICT was assessed in 1 of 2 studies (50%) [30] but was not generally accepted by users. It was unclear whether the quality of the ICTs was assessed and deemed appropriate in the remaining studies.

The overall methodological quality of included studies was strong for the website studies, while it was uncertain for the electronic education game, email, and multifaceted studies (Multimedia Appendix 4). Studies using computer software, Web-based workshops, and CDSSs were of variable methodological quality, as some studies were predominantly strong, while others were of uncertain quality. Several studies were conducted more than 10 years ago; thus, these ICTs may not reflect current technology and may no longer be relevant. The goal of this systematic review was to transparently present the current state of knowledge about ICT use among health professionals and to allow readers to make informed decisions regarding their relevance.

### Conclusion

The findings of this systematic review suggest that health professionals’ perceived usability and practice behavior change vary by type of ICT. Website studies demonstrated improvements in perceived usefulness and perceived ease of use, but not for knowledge usability, barriers, and intentions. Computer software studies demonstrated improvements in perceived usefulness, but not in knowledge and skills. Web-based workshop and email studies demonstrated improvements in knowledge, perceived usefulness, and skills. An electronic educational game intervention demonstrated an improvement in knowledge from baseline to 12 or 24 weeks. CDSS studies demonstrated variable findings for improvement in skills. Multifaceted ICT interventions demonstrated improvements in beliefs about capabilities, but not in usability. Most multifaceted ICT studies demonstrated improvements in knowledge, perceived usefulness, perceived ease of use, and beliefs about capabilities. In summary, heterogeneity and the paucity of properly conducted studies did not allow for a clear comparison between studies and a conclusion on the effectiveness of ICTs as a KT strategy for the dissemination of CPGs.

## Appendix

Multimedia Appendix 1 Search strategy.

Multimedia Appendix 2 List of excluded studies.

Multimedia Appendix 3 Included study characteristics.

Multimedia Appendix 4 Methodological assessment of included studies.

## Abbreviations

ADHD: attention-deficit/hyperactivity disorder
CDSS: computerized decision support system
CME: continuing medical education
CPG: clinical practice guideline
EHR: electronic health record
ICT: information and communication technologies
IQR: interquartile range
KT: knowledge translation
PICOS: population, intervention, comparison, outcome, and study design
PRISMA: Preferred Reporting Items for Systematic Reviews and Meta-Analyses
OR: odds ratio
TAM2: technology acceptance model
TDF: theoretical domains framework
