# Supplementary material for: Information and Communication Technologies for the Dissemination of Clinical Practice Guidelines to Health Professionals: A Systematic Review
Source: JMIR Med Educ. 2016 Nov 30;2(2):e16. doi: 10.2196/mededu.6288 (PMC5156823; doi:10.2196/mededu.6288)
Supplement: Multimedia Appendix 1 [file mededu_v2i2e16_app1.pdf]

MEDLINE
Database: Ovid MEDLINE(R) In-Process & Other Non-Indexed Citations and Ovid MEDLINE(R)
Search Strategy:
----
1 local area networks/
2 exp telemedicine/
3 (telemedicine or "tele health").tw.
4 computer communication networks/
5 internet/
6 blogging/
7 social media/
8 electronic mail/
9 search engine/
10 ("remote communication*" or "remote consultation*").tw.
11 information services/
12 (ehealth or e-health or m-health or mhealth or "health informatics").tw.
13 (internet or email or www or "world wide web" or virtual or "web site" or website).tw.
14 (e-learning or elearning or telecommunicat$).tw.
15 databases, bibliographic/
16 health information exchange/
17 libraries, digital/
18 ("computerised reminder$" or "computerized reminder$").tw.
19 ("social media*" or "second life" or facebook* or youtube or twitter* or tweet* or webmd or linkedin or noodle or zotero or mendeley or refworks or endnote or picsearch or flickr or Skype or elluminate or upstream).tw.
20 1 or 2 or 3 or 4 or 5 or 6 or 7 or 8 or 9 or 10 or 11 or 12 or 13 or 14 or 15 or 16 or 17 or 18 or 19
21 (clinician* or dentist* or doctor* or family practition* or general practition* or physician* or gyn?ecologist* or h?ematologist* or internist* or nurse* or obstetrician* or occupational therapist* or p?ediatrician* or pharmacist* or physiotherapist* or psychiatrist* or psychologist* or radiologist* or surgeon* or therapist* or counsel?or* or neurologist* or optometrist* or "health profession*” or health* personnel or “health care profession*” OR “health care personnel" or resident*).ti,ab.
22 exp evidence-based medicine/
23 clinical competence/
24 professional competence/
25 professional practice/
26 guideline adherence/
27 guidelines as topic/
28 22 or 23 or 24 or 25 or 26 or 27
29 randomized controlled trial.pt.
30 controlled clinical trial.pt.
31 randomized.ab.
32 placebo.ab.
33 clinical trials as topic.sh.
34 randomly.ab.
35 trial.ti.
36 (control* adj8 trial*).ti,ab.
37 (systematic: review or systematic: overview).ti,ab. or meta-analysis.pt,sh. or (meta-anal: or metaanal:).tw.
38 29 or 30 or 31 or 32 or 33 or 34 or 35 or 36 or 37

39 exp animals/ not humans.sh.
40 38 not 39
41 20 and 21 and 28 and 40

****

Database: Ovid MEDLINE(R) In-Process & Other Non-Indexed Citations and Ovid MEDLINE(R)
Search Strategy:
----
1 (telemedicine or "tele health").tw.
2 ("remote communication*" or "remote consultation*").tw.
3 (ehealth or e-health or m-health or mhealth or "health informatics").tw.
4 (internet or email or www or "world wide web" or virtual or "web site" or website).tw.
5 (e-learning or elearning or telecommunicat$).tw.
6 ("digital librar*" or "electronic librar*").tw.
7 ("computerised reminder$" or "computerized reminder$").tw.
8 ("social media*" or "second life" or facebook* or youtube or twitter* or tweet* or webmd or linkedin or noodle or zotero or mendeley or refworks or endnote or picsearch or flickr or Skype or elluminate or upstream).tw.
9 email or internet or social media).ti,ab.
10 1 or 2 or 3 or 4 or 5 or 6 or 7 or 8 or 9
11 ("clinical competence" or evidence-based or "professional competence" or guideline*).ti,ab.
12 (randomized or placebo or randomly).ab. or trial.ti. or (control* adj8 trial*).ti,ab. or (systematic: review or systematic: overview).ti,ab. or (meta-anal: or metaanal:).tw.
13 10 and 11 and 12
14 limit 13 to in process

****

COCHRANE CENTRAL
Database: EBM Reviews - Cochrane Central Register of Controlled Trials
Search Strategy:
----
1 (clinician* or dentist* or doctor* or "family practition*" or "general practition*" or physician* or gyn?ecologist* or h?ematologist* or internist* or nurse* or obstetrician* or "occupational therapist*" or p?ediatrician* or pharmacist* or physiotherapist* or psychiatrist* or psychologist* or radiologist* or surgeon* or therapist* or counsel?or* or neurologist* or optometrist* or "health* professional*" or health* personnel or "health care professional*" OR "health care personnel" or resident*).ti,ab.
2 ("clinical practic*" or "clinical competenc*" or "professional practice" or "professional competenc*" or "guideline* adj3 adherence" or "evidence-based" or "evidence based").ti,ab.
3 (phone* or texting or email* or email* or MSN* or SMS* or pda or "personal digital assistant*" or "smart phone*" or smartphone* or i-phone* or i phone* or tablet or computer* or internet or "information communication technolog*" or online or Virtual or "world wide web*" or "social media*" or "second life" or facebook* or youtube or twitter* or tweet* or webmd or linkedin or noodle or zotero or mendeley or refworks or endnote or picsearch or flicker or blog* or wiki or podcast* or "RSS feed*" or "really simple syndicat*" or "video conferenc*" or teleconference* or "real-time app*" or "real time app*" or Skype or illuminate or stream or "digital librar*" or "electronic librar*" or "electronic database*").ti,ab.
4 1 and 2 and 3

****

EMBASE
Database: Embase Classic+Embase
Search Strategy:

----

1 local area network/
2 exp telehealth/
3 (telemedicine or "tele health").tw.
4 computer network/
5 internet/
6 social media/
7 e-mail/
8 search engine/
9 ("remote communication*" or "remote consultation*").tw.
10 information service/
11 (health or e-health or m-health or mhealth or "health informatics").tw.
12 (internet or email or www or "world wide web" or virtual or "web site" or website).tw.
13 (e-learning or elearning or telecommunicat$).tw.
14 exp bibliographic database/
15 ("digital librar*" or "electronic librar*").tw.
16 ("computerised reminder$" or "computerized reminder$").tw.
17 ("social media*" or "second life" or facebook* or youtube or twitter* or tweet* or webmd or linkedin or noodle or zotero or mendeley or refworks or endnote or picsearch or flickr or Skype or elluminate or upstream).tw.
18 1 or 2 or 3 or 4 or 5 or 6 or 7 or 8 or 9 or 10 or 11 or 12 or 13 or 14 or 15 or 16 or 17
19 (clinician* or dentist* or doctor* or family practition* or general practition* or physician* or gyn?ecologist* or h?ematologist* or internist* or nurse* or obstetrician* or occupational therapist* or p?ediatrician* or pharmacist* or physiotherapist* or psychiatrist* or psychologist* or radiologist* or surgeon* or therapist* or counsel?or* or neurologist* or optometrist* or "health profession*” or health* personnel or “health care profession*” OR “health care personnel" or resident*).ti,ab.
20 *evidence based medicine/ or *evidence based practice/ or *evidence based nursing/ or *evidence based emergency medicine/ or *evidence based dentistry/ or *evidence based practice center/
21 *clinical competence/
22 exp *professional competence/
23 *good clinical practice/
24 *practice guideline/
25 20 or 21 or 22 or 23 or 24
26 controlled clinical trial/
27 randomized controlled trial/
28 randomized.ab.
29 placebo.ab.
30 randomly.ab.
31 trial.ti.
32 (control* adj8 trial*).ti,ab.
33 (systematic: review or systematic: overview).ti,ab. or meta-analysis.pt,sh. or (meta-anal: or metaanal:).tw.
34 26 or 27 or 28 or 29 or 30 or 31 or 32 or 33
35 18 and 19 and 25 and 34

****

PsycINFO

Database: PsycINFO

Search Strategy:

----

1 internet/

2 computer mediated communication/

3 cellular phones/

4 electronic communication/

5 social media/

6 computer searching/

7 mobile devices/

8 telemedicine/

9 teleconferencing/

10 (telemedicine or "tele health").tw.

11 online social networks/

12 ("remote communication*" or "remote consultation*").tw.

13 (ehealth or e-health or m-health or mhealth or "health informatics").tw.

14 (internet or email or www or "world wide web" or virtual or "web site" or website).tw.

15 (e-learning or elearning or telecommunicat$).tw.

16 automated information retrieval/

17 computer searching/

18 information technology/

19 ("digital librar*" or "electronic librar*").tw.

20 ("computerised reminder$" or "computerized reminder$").tw.

21 ("social media*" or "second life" or facebook* or youtube or twitter* or tweet* or webmd or linkedin or noodle or zotero or mendeley or refworks or endnote or picsearch or flickr or Skype or elluminate or upstream).tw.

22 1 or 2 or 3 or 4 or 5 or 6 or 7 or 8 or 9 or 10 or 11 or 12 or 13 or 14 or 15 or 16 or 17 or 18 or 19 or 20 or 21

23 (clinician* or dentist* or doctor* or family practition* or general practition* or physician* or gyn?ecologist* or h?ematologist* or internist* or nurse* or obstetrician* or occupational therapist* or p?ediatrician* or pharmacist* or physiotherapist* or psychiatrist* or psychologist* or radiologist* or surgeon* or therapist* or counsel?or* or neurologist* or optometrist* or "health profession*" or health* personnel or "health care profession*" OR "health care personnel" or resident*).ti,ab.

24 clinical practice/

25 professional competence/

26 treatment guidelines/

27 evidence based practice/

28 24 or 25 or 26 or 27

29 randomized.ab.

30 placebo.ab.

31 randomly.ab.

32 trial.ti.

33 (control* adj8 trial*).ti,ab.

34 (systematic: review or systematic: overview).ti,ab. or meta-analysis.pt,sh. or (meta-anal: or metaanal:).tw.

35 29 or 30 or 31 or 32 or 33 or 34

36 22 and 23 and 28 and 35

****
