# Supplementary material for: Information and Communication Technologies for the Dissemination of Clinical Practice Guidelines to Health Professionals: A Systematic Review
Source: JMIR Med Educ. 2016 Nov 30;2(2):e16. doi: 10.2196/mededu.6288 (PMC5156823; doi:10.2196/mededu.6288)
Supplement: Multimedia Appendix 2 [file mededu_v2i2e16_app2.pdf]

Study	Reason for exclusion
Bailey TC, Noirot LA, Blickensderfer A, Rachmiel E, Schaiff R, Kessels A, et al. An intervention to improve secondary prevention of coronary heart disease. Arch Intern Med. 2007; 167(6): 586-90.	Irrelevant population
Coberly E, Boren SA, Mittal M, Davis JW, Scoville C, Chitima-Matsiga R et al. MedlinePlus-based health information prescriptions: A comparison of email vs paper delivery. Inform Prim Care. 2012; 20(3): 197-205	Irrelevant intervention
Buchanan H, Siegfried N, Jelsma J, Lombard C. Comparison of an interactive with a didactic educational intervention for improving the evidence-based practice knowledge of occupational therapists in the public health sector in south Africa: A randomised controlled trial. Trials. 2014; 15:216 doi: 10.1186/1745-6215-15-216.	Irrelevant intervention
Bury J, Hurt C, Roy A, Cheesman L, Bradburn M, Cross S, et al. LISA: A web-based decision-support system for trial management of childhood acute lymphoblastic leukaemia. British Journal of Haematology.2005; 129(6), 746-54.	Irrelevant intervention
Bunt CW, Burke HB, Towbin AJ, Goang A, Stephens MB, Gontelo P et al. Point-of-care estimated radiation exposure and imaging guidelines can reduce pediatric radiation burden. Journal of the American Board of Family Medicine. 2015; 28(3): 343-350	Irrelevant outcome
Campbell L, Novak I, McIntyre S. Patterns and rates of use of an evidence-based practice intranet resource for allied health professionals: a randomised controlled trial. Developmental Medicine and Child Neurology. 2010; 52(S2):31.	Abstract
Campbell L, Novak I, McIntyre S. Effectiveness of providing evidence-based practice education with workplace supports for changing health professionals decision-making and outcomes of care: an evaluator-blinded randomised controlled trial. Aust Occup Ther J. 2011; 58(S1): 120.	Abstract
Campbell L, Novak I, McIntyre S, Lord S. A KT intervention including the evidence alert system to improve clinician's evidence-based practice behavior—a cluster randomized controlled trial. Implement Sci. 2013; 13; 8: 132.	Irrelevant intervention
Cannon DS, Allen SN. A comparison of the effects of computer and manual reminders on compliance with a mental health clinical practice guideline. Journal of the American Medical Informatics Association. 2000; 7(2): 196-203.	Irrelevant population
Caulfield JJ. Examining the effect of teaching method and learning style on work performance for practicing home care clinicians. ProQuest Dissertations and Theses.2001; 143-143 (275860831).	Irrelevant intervention
Chan D, Patel P, Booth L, Lee D, Dent T, Harris F et al. A novel approach for implementing evidence-based guidelines in the community: The appropriate choices in dyspepsia project. Journal of Clinical Excellence. 2001; 2(4): 219-24.	Irrelevant intervention
Choi J. Development and Evaluation of a Computer-Interpretable Guideline for Depression Screening and Initial Management in Primary Care [dissertation]. New York, NY: Columbia University. 2006; 195-195.	Inappropriate study design
Cicolini G, Simonetti V, Comparcini D, Celiberti I, Di Nicola M, Capasso LM et al. Efficacy of a nurse-led email reminder program for cardiovascular prevention risk reduction in hypertensive patients: A randomized controlled trial. International journal of nursing studies. 2014; 51(6): 833-43.	Irrelevant population
Davis J, Chryssafidou E, Zamora J, Davies D, Khan K, Coomarasamy A. Computer-based teaching is as good as face to face lecture-based teaching of evidence based medicine: a randomised controlled trial. BMC medical education. 2007; 7(1): 23.	Irrelevant intervention
Durieux P, Nizard R, Ravaud P, Mounier N, Lepage E. A clinical decision support system for prevention of venous thromboembolism: effect on physician behavior. Jama. 2000; 283(21): 2816-21.	Irrelevant outcome

Eaton CB, Parker DR, Borkan J, McMurray J, Roberts MB, Lu B et al. Translating cholesterol guidelines into primary care practice: a multimodal cluster randomized trial. The Annals of Family Medicine. 2011; 9(6): 528-37.	Irrelevant outcome
Eccles M, McColl E, Steen N, Rousseau N, Grimshaw J, Parkin D et al. Effect of computerised evidence based guidelines on management of asthma and angina in adults in primary care: cluster randomised controlled trial. BMJ. 2002; 325(7370): 941.	Irrelevant outcome
Etxeberria A, Pérez I, Alcorta I, Emparanza JI, de Velasco ER, Iglesias MT et al. The CLUES study: a cluster randomized clinical trial for the evaluation of cardiovascular guideline implementation in primary care. BMC health services research. 2013; 13(1):1.	Protocol
Farah SS, Winter M, Appu S. Helping doctors utilize the prostate□specific antigen effectively: an online randomized controlled trial (The DUPE trial). ANZ journal of surgery. 2012; 82(9): 633-8.	Irrelevant intervention
Fricton J, Rindal DB, Rush W, Flottemesch T, Vazquez G, Thoele MJ et al. The effect of electronic health records on the use of clinical care guidelines for patients with medically complex conditions. The Journal of the American Dental Association. 2011; 142(10): 1133-42.	Irrelevant outcome
Gerbert B, Bronstone A, Maurer T, Berger T, McPhee SJ, Caspers N. The effectiveness of an internet□based tutorial in improving primary care physicians' skin cancer triage skills. Journal of Cancer Education. 2002; 17(1):7-11.	Irrelevant intervention
Gordon M, Chandratilake M, Baker P. Improved junior paediatric prescribing skills after a short e-learning intervention: a randomised controlled trial. Archives of disease in childhood. 2011; 96(12):1191-4.	Irrelevant intervention
Ista E, van Dijk M, van Achterberg T. Do implementation strategies increase adherence to pain assessment in hospitals? A systematic review. International journal of nursing studies. 2013; 50(4):552-68.	Irrelevant intervention
Korner-Bitensky N, Roy MA, Teasell R, Kloda L, Storr C, Asseraf-Pasin L et al. Creation and pilot testing of StrokEngine: a stroke rehabilitation intervention website for clinicians and families. Journal of rehabilitation medicine. 2008; 40(5):329-33.	Irrelevant intervention
Laibhen-Parkes N, Codone S. Web-based evidence based practice educational intervention to improve EBP competence among BSN-prepared pediatric bedside nurses: a mixed methods pilot study. Journal of Nursing. 2014;1(1):2.	Irrelevant intervention
Liaw SY, Wong LF, Chan SW, Ho JT, Mordiffi SZ, Ang SB, Goh PS, Ang EN. Designing and Evaluating an Interactive Multimedia Web-Based Simulation for Developing Nurses' Competencies in Acute Nursing Care: Randomized Controlled Trial. Journal of medical Internet research. 2015; 17(1).	Irrelevant intervention
Lee NJ, Bakken S. Development of a prototype personal digital assistant-decision support system for the management of adult obesity. International journal of medical informatics. 2007; 76:S281-92.	Irrelevant population
Lobach DF, Hammond WE. Computerized decision support based on a clinical practice guideline improves compliance with care standards. The American journal of medicine. 1997; 102(1):89-98.	Irrelevant intervention
Murtaugh CM, Pezzin LE, McDonald MV, Feldman PH, Peng TR. Just□in□Time Evidence□Based E□mail "Reminders" in Home Health Care: Impact on Nurse Practices. Health Services Research. 2005; 40(3): 849-64.	Duplicate
Nilasena DS, Lincoln MJ. A computer-generated reminder system improves physician compliance with diabetes preventive care guidelines. InProceedings of the Annual Symposium on Computer Application in Medical Care 1995 (p. 640).	Irrelevant population

American Medical Informatics Association.	
Noonan VK, Wolfe DL, Thorogood NP, Park SE, Hsieh JT, Eng JJ. Knowledge translation and implementation in spinal cord injury: a systematic review. Spinal cord. 2014; 52(8):578-87.	Irrelevant intervention
Pearce J, Mann MK, Jones C, van Buschbach S, Olff M, Bisson JI. The most effective way of delivering a Train□the□Trainers program: A systematic review. Journal of Continuing Education in the Health Professions. 2012; 32(3):215-26.	Irrelevant outcome
Putten GJ, Visschere L, Schols J, Baat C, Vanobbergen J. Supervised versus non-supervised implementation of an oral health care guideline in (residential) care homes: a cluster randomized controlled clinical trial. BMC oral health. 2010; 10(1):1.	Irrelevant intervention
Raghu A, Praveen D, Peiris D, Tarassenko L, Clifford G. Engineering a mobile health tool for resource-poor settings to assess and manage cardiovascular disease risk: SMARThealth study. BMC medical informatics and decision making. 2015; 15(1): 1.	Inappropriate study design
Stein BD, Celedonia KL, Swartz HA, DeRosier ME, Sorbero MJ, Brindley RA et al. Implementing a web-baed intervention to train community clinicians in an evidence-based psychotherapy: a pilot study. Psychiatric Services. 2015.	Irrelevant intervention
Thomas L, Cullum N, McColl E, Rousseau N, Soutter J, Steen N. Guidelines in professions allied to medicine. The Cochrane Database of Systematic Reviews. 1999; 1.	Irrelevant intervention
Tierney WM, Overhage JM, Murray MD, Harris LE, Zhou XH, Eckert GJ et al. Effects of computerized guidelines for managing heart disease in primary care. Journal of General Internal Medicine. 2003; 18(12): 967-76.	Irrelevant population
Tierney WM, Overhage JM, Murray MD, Harris LE, Zhou XH, Eckert GJ et al. Can Computer□Generated Evidence□Based Care Suggestions Enhance Evidence□Based Management of Asthma and Chronic Obstructive Pulmonary Disease? A Randomized, Controlled Trial. Health services research. 2005; 40(2): 477-98.	Irrelevant population
Vollmar HC, Butzlaff ME, Lefering R, Rieger MA. Knowledge translation on dementia: a cluster randomized trial to compare a blended learning approach with a. BMC health services research. 2007; 7(1):92.	Protocol
Were MC, Shen C, Tierney WM, Mamlin JJ, Biondich PG, Li X, et al. Evaluation of computer-generated reminders to improve CD4 laboratory monitoring in sub-Saharan Africa: a prospective comparative study. Journal of the American Medical Informatics Association. 2011; 18(2): 150-5.	Irrelevant population
