# Supplementary material for: Information and Communication Technologies for the Dissemination of Clinical Practice Guidelines to Health Professionals: A Systematic Review
Source: JMIR Med Educ. 2016 Nov 30;2(2):e16. doi: 10.2196/mededu.6288 (PMC5156823; doi:10.2196/mededu.6288)
Supplement: Multimedia Appendix 3 [file mededu_v2i2e16_app3.pdf]

Study	Country	Study Design	Population	Intervention	Comparator	Outcome(s) (Framework)	Duration/ Follow-up
Balamuth et al. [22]	US	RCT	physicians	Online 1-page summary sheet of guidelines (n=128)	Web link to guidelines (n=109)	Knowledge (TDF)<br><br>Perceived Ease of Use (TAM2)	6 weeks
Bell et al. [23]	US	RCT	family and internal medicine residents	Self-study online guidelines (n=79)	Print-based guidelines (n=83)	Knowledge (TDF)<br><br>Perceived Ease of Use (TAM2)	immediate post-intervention, 4 to 6 months
Bernhardsson et al. [33]	Sweden	CCT	physiotherapists	Multifaceted: Implementation seminar/group discussion, website and email reminders (n=168)	No intervention (n=88)	Knowledge (TDF)<br><br>Perceived Ease of Use (TAM2)	12 months
Bullard et al. [26]	Canada	RCT	physicians	Wirelessly networked mobile computer program (n=10)**	Desktop computer program (n=10)**	Perceived Usefulness (TAM2)	8-hour shifts/3 months
Butzlaff et al. [27]	Germany	RCT	physicians	CPGs via CD-ROM/Interne (n=53)	No intervention (n=66)	Knowledge (TDF)	~70 days, ~5 months
Chan et al. [34]	Canada	RCT	nurses	Multifaceted in-person education session and online support (n=31)	No intervention (n=22)	Beliefs about capabilities (TDF)<br><br>Intention (TDF)<br><br>Perceived Usefulness (TAM2)	2 weeks
Desimone et al. [35]	US	RCT	internal medicine residents	Multifaceted: In person education, online support, printed material (n=11)	Usual education (n=11)	Knowledge (TDF)	4 weeks
Epstein et al. [20]	US	RCT	physicians (pediatricians)	Online didactic education session/workshop (n=27)	No intervention (received intervention after 6 months) (n=22)	Skills (TDF)	6 months
Fordis et al. [29]	US	RCT	physicians	Live online CME workshop (n=51)<br><br>Online CME workshop (n=52)	No intervention (n=20)	Knowledge (TDF)<br><br>Skills (TDF)<br><br>Perceived Usefulness (TAM2)	12 weeks
Gill et al. [30]	US	RCT	physicians/clinicians in ambulatory practices	EHR-based clinical decision support (n=53	No intervention (n=66)	Skills (TDF)	12 months
Schroter et al. [17]	UK/Germany	RCT	physicians/nurses	Interactive web-based tool + online material (n=527)	Online material (n=527)	Knowledge (TDF)<br><br>Perceived Usefulness (TAM2)	4 months
Jousimaa et al. [28]	Finland	RCT	physicians	CD-ROM computer-based guidelines (n=72)	Text-book-based guidelines (n=67)	Skills (TDF)	1 month
Kerfoot et al. [21]	International*	RCT	physicians (urologists)	Online game/survey 2 questions every 2 days (n=735)	Online game/survey 4 questions every 4 days (n=735)	Knowledge (TDF)	34 weeks
Lobach et al. [19]	US	RCT	physicians /general internist/nurses/ph	Biweekly email of computer based audit/feedback	No intervention (n=23)	Skills (TDF)	12 weeks

			ysician assistant/family medicine residents	program (n=22)			
McDonald et al. [36]	UK	RCT	nurses	Multifaceted: Email reminder with provider prompts, patient education material, and clinical nurse specialist outreach (n=97)<br><br>Email reminder of recommendations (n=121)	Usual care (n=118)	Skills (TDF)	24 months
Fretheim et al. [18]	US	RCT	physicians and practice nurses	Multifaceted: Educational Outreach Visit, Audit and Feedback at Outreach Visit, Computerized Reminders , Risk Assessment Tools, Patient Information Material, Telephone follow-up (n=257)	Passive guideline dissemination (n=244)	Skills (TDF)	45 days
Peremans et al. [31]	France	RCT	nurses	EHR-based clinical decision support (n=15)<br><br>Empowered patient group (n=15)	No intervention (n=13)	Skills (TDF)	24 weeks (10 week intervention, 4 week washout, 10 week control)
Sassen et al. [24]	Norway	RCT	physicians (orthopedic surgeons)	Website with educational modules (n=48)	Waiting list (n=33)	Intention (TDF)<br><br>Barriers (TDF)	12 months
Shenoy [37]	Belgium	RCT	physicians	Multifaceted: education, audit, feedback (n=24)	Mailed guidelines (n=21)	Knowledge	5 months
Stewart et al. [32]	Netherlands	RCT	physicians	Email on-line learning for two evidence-based cases (type 2 diabetes, prevention) (n=27)	Waiting list (n=31)	Knowledge (TDF)<br><br>Skills (TDF)	6 months
Wolpin et al. [25]	US	RCT	medicine residents and fellows	Website enhanced learning (additional case studies) (n=33)	Website with usual care instructions (same content, without case studies) (n=36)	Knowledge (TDF)<br><br>Perceived Ease of Use (TAM2)	12 weeks

*A total of 63 countries were included. Please see publication for complete list.
** cross-over design
CCTs = comparative controlled trials; CD-ROM = compact disc read-only memory; CDSS = computerized decision support system; CME = continuing medical education; CI = confidence interval; EHR = electronic health record; IQR = interquartile range (25th and 75th percentile); NR = not reported; OR =

odds ratio; RCT = randomized controlled trial; TAM2 = Technology Acceptance Model; TDF = Theoretical Domains Framework; UK = United Kingdom; US = United States of America
