# Supplementary material for: Information and Communication Technologies for the Dissemination of Clinical Practice Guidelines to Health Professionals: A Systematic Review
Source: JMIR Med Educ. 2016 Nov 30;2(2):e16. doi: 10.2196/mededu.6288 (PMC5156823; doi:10.2196/mededu.6288)
Supplement: Multimedia Appendix 4 [file mededu_v2i2e16_app4.pdf]

Study	Random sequence generation	Allocation concealment	Blinding of participants and personel	Blinding of outcome assessment	Incomplete outcome data	Selective reporting	Other Bias
Balamuth et al. [22]	+	?	-	?	+	+	-
Bell et al. [23]	+	?	-	?	+	+	+
Bernhardsson et al. [33]	-	?	?	?	-	+	+
Bullard et al. [26]	+	-	-	?	+	+	-
Butzlaff et al. [27]	+	+	?	?	+	+	+
Chan et al. [34]	?	?	?	?	-	+	-
Desimone et al. [35]	?	?	-	?	?	+	-
Epstein et al. [20]	+	?	-	?	+	?	-
Gill et al. [30]	?	?	?	?	-	+	-
Schroter et al. [17]	+	+	?	?	-	+	+
Jousimaa et al. [28]	+	?	?	?	+	?	+
Kerfoot et al. [21]	+	?	?	?	-	?	+
Fordis et al. [29]	+	?	?	+	+	+	-
Lobach et al. [19]	-	?	?	?	?	+	-
McDonald et al. [36]	+	?	?	+	-	?	-
Fretheim et al. [18]	+	+	+	+	+	+	+
Peremans et al. [31]	+	?	+	?	+	?	+
Sassen et al. [24]	+	?	?	?	-	+	-
Shenoy [37]	?	?	?	?	+	+	-
Stewart et al. [32]	+	?	?	?	-	?	-
Wolpin et al. [25]	-	-	?	?	-	?	-

+ = low risk of bias; - = high risk of bias; ? = uncertain risk of bias
